# A 3D-printed capillary tube holder for high-throughput chemotaxis assays

**DOI:** 10.1128/jb.00384-25

**Published:** 2025-12-18

**Authors:** Chiara Berruto, Elisa Grillo, Shrila Esturi, Gozde S. Demirer

**Affiliations:** 1 Biology and Biological Engineering Division, California Institute of Technology 6469, Pasadena, California, USA; 2 Chemistry and Chemical Engineering Division, California Institute of Technology 6469, Pasadena, California, USA; University of Massachusetts Chan Medical School, Worcester, Massachusetts, USA

**Keywords:** bacterial chemotaxis, high-throughput assay, chemoattractant screening, capillary tube, chemoeffectors

## Abstract

Bacterial chemotaxis is an important behavior to study to understand spatial segregation of species in mixed communities and the assembly of host microbiomes. This is particularly relevant in the rhizosphere, where chemoattraction toward root exudates is an important determinant of plant colonization. However, current methods to screen chemoeffectors are limited in their throughput, creating a barrier to generating comprehensive data sets describing chemotactic profiles for species of interest. Here, we describe a novel 3D-printed capillary tube holder approach, which facilitates up to 384 simultaneous capillary tube chemotaxis assays. We optimized and benchmarked our assay using *Escherichia coli* K12 and *Bacillus subtilis* 3610 with known chemoattractants: serine and aspartate. We then tested the threshold concentration of these chemoattractants using our assay and found that we could detect chemoattraction toward concentrations spanning multiple orders of magnitude. In this paper, we describe our high-throughput chemotaxis assay in detail and provide the necessary files for 3D printing the capillary tube holder.

**IMPORTANCE:**

Chemotaxis is an important behavior to study to understand how microbial communities assemble and respond to their environment. Identifying chemoattractants may uncover key targets for microbiome engineering. However, the generation of large data sets describing chemotactic profiles has been limited by a lack of high-throughput tools to quantitatively screen chemotaxis. We designed a 3D-printed assay allowing for up to 384 simultaneous capillary tube chemotaxis assays and validated our method with two different bacterial species. The throughput of our approach is greatly increased by the ability to use lag time as a proxy for cell count. Our approach is easy to use and low cost, effectively lowering the barrier to expanding more comprehensive data sets describing the chemotactic profiles of different bacterial species.

## INTRODUCTION

Chemotaxis is the process by which motile organisms direct their movement along chemical gradients. Chemotaxis may be toward (chemoattraction) or away from (chemorepulsion) a given chemical signal facilitating the migration of cells to desirable environments (1, 2). Bacteria utilize different forms of motility to carry out chemotaxis, including flagella-based swimming and pili-mediated twitching or swarming (1, 2). This directed motility allows bacteria to forage for nutrients, travel toward hosts or symbionts, and create spatial segregation in microbial communities (1, 2). This makes chemotaxis an important phenomenon to study to understand host-microbe interactions and ecological behavior.

Various tools have been developed to study bacterial chemotaxis, each having their own advantages and disadvantages (3–6). Historically, capillary tube assays have been popular due to the low cost and ease of use. Capillary tube assays can be either quantitative or qualitative depending on the method (6). Qualitative capillary tube assays involve observing bacterial cell accumulation at the end of a capillary tube containing an attractant using darkfield microscopy (6). These assays can test whether different chemicals are chemoattractants but do not provide adequate information to compare the efficacy of different chemoeffectors. For this, quantitative measurements are necessary. The quantitative capillary tube assay involves incubating a capillary tube containing a chemoeffector in a reservoir of bacteria and measuring how many cells swim into the capillary tube via colony-forming unit (CFU) counting (7). This number is then compared to the number of cells found in a buffer control capillary tube. This assay is both quantitative and highly accessible for researchers, making it a popular tool to investigate bacterial chemotactic responses to different chemicals. However, the assay is low throughput and requires precise handling of small volumes of liquids. These barriers make it challenging and time intensive to screen many different chemoeffectors, limiting the quantity and quality of available data sets describing bacterial chemotactic profiles. A high-throughput, quantitative, reproducible, and low-cost method is still needed to enable researchers to better characterize chemotactic profiles and generate more comprehensive data sets for bacterial species of interest.

Here, we describe a high-throughput chemotaxis assay using a 3D-printed capillary tube holder that enables simultaneous screening of up to 384 chemoeffectors without the need for CFU counting. Our design is inspired by a prior approach showing that chemotaxis assays can be carried out in a plate-based fashion using lag time as a proxy for cell count (8). We substantially improved upon this approach, replacing the Whatman UniFilter plates with a customizable 3D-printed capillary holder, effectively decreasing the cost and increasing the accessibility and throughput of the assay. We describe capillary tube holder designs of 96- and 384-well formats; however, they can easily be adapted to fit other desired plate layouts or capillary tube dimensions. To validate our method, we used previously characterized chemoattractants for *Escherichia coli* K12 (9) and *Bacillus subtilis* 3610 (10). The chemotactic behavior of these two species has been extensively characterized using traditional capillary tube assays, allowing us to optimize our design and benchmark our results (9, 10). We chose these two species to demonstrate that our design can be applied to both gram-negative (*E. coli*) and gram-positive (*B. subtilis*) species and broadly to species with different ecological relevance.

## RESULTS

### Design and optimization of the 3D-printed capillary tube holder

**Fig 1:**
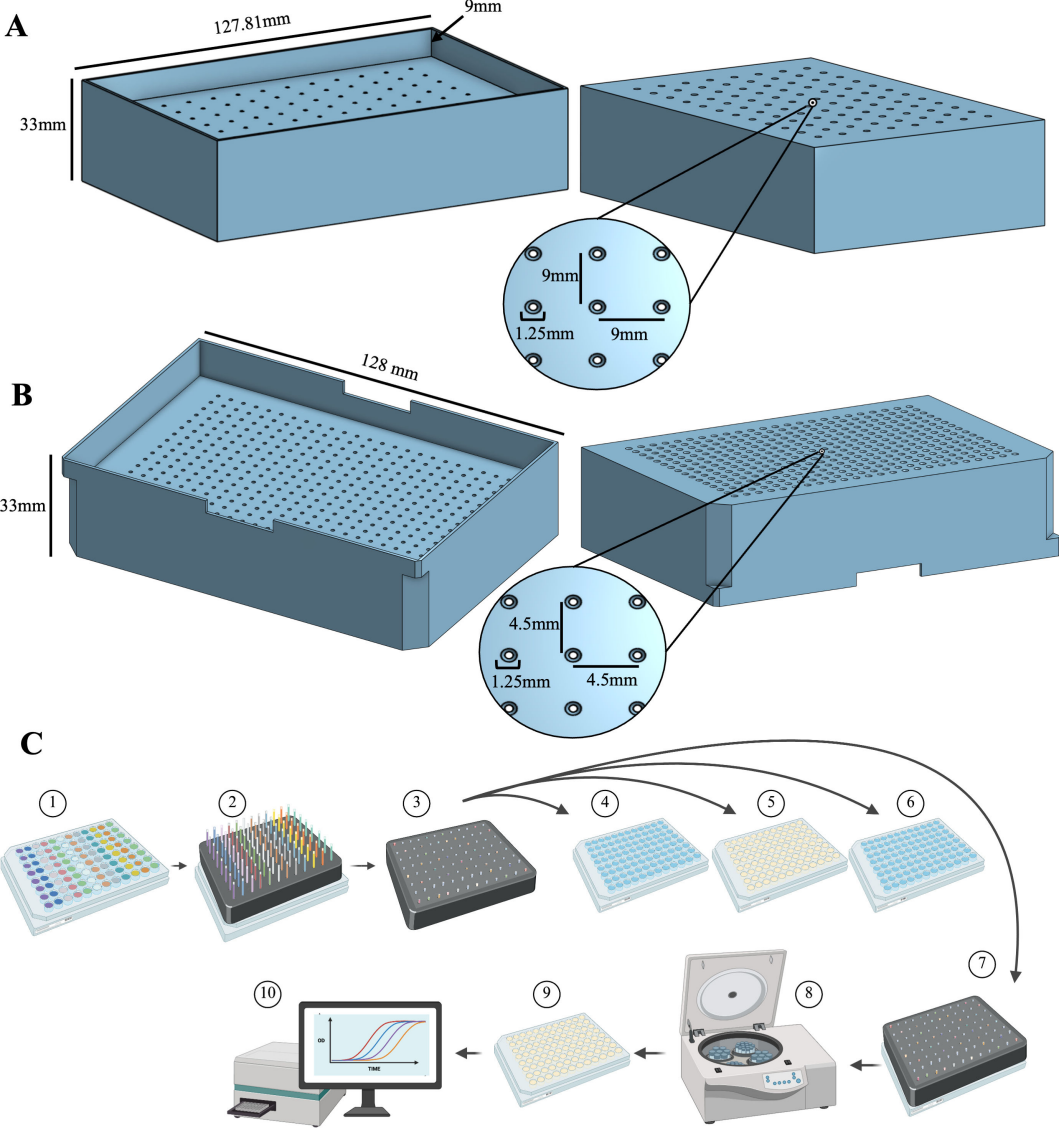
High-throughput chemotaxis assay overview. Bottom (left) and top (right) views of the (**A**) 96-well and (**B**) 384-well capillary holders, including relevant measurements. (**C**). Chemotaxis assay method schematic: (1) Chemoeffector plate is filled with chemoeffectors to be tested in the desired layout; (2) capillary holder is filled with capillaries and placed on top of the chemoeffector plate until capillaries fill with chemoeffectors; (3) top of the capillary holder is sealed with plate-sealing film; (4) capillary holder is dipped in the wash plate; (5) capillary holder is transferred to bacterial plate and incubated for 45 min; (6) capillary holder is dipped in wash plate; (7) capillary holder is moved to an empty plate; (8) capillary holder on the empty plate is spun down to expel capillaries; (9) the capillary holder is removed, and growth media are added to the plate; and (10) growth curve data are collected in a plate reader.

Our 3D-printed capillary holder facilitates plate-based screening of chemoattractants and is designed to hold one capillary tube per well. The dimensions of the 96-well and 384-well capillary tube holders are shown in Fig. 1A and B, respectively. For our assays, we used Fisherbrand 96-Well plates and Nunc 384-Well plates, but the 3D-printed capillary holder is compatible with well plates of similar dimensions. The capillary holder design accommodates 1 µL capillary tubes. The holder is designed to accommodate the full length of the capillary tube, holding it in the well such that the top of the capillary is flush with the top of the holder, and the bottom of the capillary is submerged in the plate liquid but not touching the bottom of the well. This ensures the opening to the capillary tube is accessible for the bacteria to swim freely and that the top of the capillaries can be easily sealed with a plate film. Use of capillary tubes with different dimensions will require altering the capillary holder design. See Materials and Methods for the specific catalog numbers of the well plates and capillary tubes used. The capillaries must fit tightly within the capillary holder to allow for proper expulsion during the centrifugation step, which we found to be greatly influenced by the filament type used in 3D printing. Printing the capillary tube holder with thermoplastic polyurethane filament (TPU) filament improved the ability of the holder to tightly hold the capillaries without breaking compared to using polylactic acid filament.

In addition to the material used, the resolution of the 3D printer and the print conditions (see Materials and Methods) influence the optimal diameter of the capillary openings in the holder, and calibration may be needed for prints made under different conditions. We included a calibration print (File S1), which can be printed to determine the optimal diameter for the holes in the capillary tube holder. More details on printing specifics are described in Materials and Methods. Design files to print the capillary tube holders and calibration piece are included as File S1 and S2; see File S3 at https://doi.org/10.5061/dryad.2jm63xt2v.

### Chemotaxis assay development

This method uses four-well plates between which the capillary tube holder with the capillary tubes is transferred during the assay. The schematic of the method is shown in Fig. 1C. First, the capillary tube holder is filled with capillaries and placed atop the chemoeffector plate. The capillaries fill with chemoeffectors from the plate via capillary action. The top of the holder is sealed with a plate film. The capillary tube holder with capillaries full of chemoeffectors is dipped into a wash plate containing sterile ddH_2_O water to wash the outside of the capillaries before transferring into the bacteria plate. The bacteria plate is filled with bacterial cells that are washed and resuspended in motility media, and the capillary holder is incubated on the bacteria plate to allow the bacterial cells to swim into the capillary tubes. At the end of the incubation period, the outside of the capillary tubes is washed by dipping into the wash plate, and the capillary tube holder carrying the capillaries is transferred to an empty plate. The seal is removed from the top of the holder, and the capillaries are expelled into the empty plate via centrifugation. Finally, the capillary holder is removed; growth media is added to each well; and the plate is placed in a plate reader to record bacterial growth.

The data generated from the growth curve can be analyzed to record the lag time of each well, which we define as the time it takes for each well to reach an OD_600_ of 0.3. The lag time is used to calculate the initial number of cells in each well using a standard curve (8). From the cell counts, a chemotaxis index (CI) can be calculated using the following equation: CI = *T* / (*T* + *C*), where *T* is the number of cells in the chemoeffector capillary, and *C* is the number of cells in the control capillary (11). CI values greater than 0.6 indicate an attractant, while CI values less than 0.4 indicate a repellent, and CI values between 0.4 and 0.6 are considered neutral (11). Alternative methods for calculating chemotaxis indexes may be used (12, 13).

Parameters to maximize sensitivity of chemotaxis assays have previously been described for both *B. subtilis* (10) and *E. coli* (7, 9). Parameters of importance include type of motility media, wash conditions, temperature of the assay, cell concentration in the reservoir, chemoeffector concentration, incubation time, and capillary size (7, 9). From previous literature, we decided to use motility media + glycerol (10 mM potassium phosphate buffer, pH 7; 0.1 mM EDTA; and 0.05% glycerol) to wash and resuspend our cells to an OD_600_ = 0.01 in the bacterial reservoir, and we used a chemoeffector concentration of 10 mM to maximize the response during assay development. Additionally, we optimized the wash protocol to maximize cell motility and chemotactic response. We found that it was critical to treat the cells very gently while washing to avoid disrupting motility machinery. This involved spinning down the cells for 5 min at 1,700 rcf before removing the supernatant, adding in motility media, and inverting to mix rather than pipette mixing to resuspend the pellet. We verified motility using microscopy and found that washing cells two times followed by a 30-min recovery period at 37°C improved motility to levels comparable to prewashed cell cultures. Washing cells two times also improved chemotactic response compared to cells washed a single time. We suspect this is due to carryover of carbon when cells are washed only once, which would disrupt chemotactic gradients. Another key parameter we optimized was the incubation time for the experiment, which we found to be optimal at 45 min. It is worth noting that the parameters may need to be reoptimized, depending on the species being tested.

### Constructing standard curves

To quantify the number of cells in each capillary tube using growth curve data, standard curves must be constructed for each species being tested. The standard curves are used to calculate the number of cells in each well from the lag time value. To construct these standard curves, twofold serial dilutions of bacteria in growth media were prepared. These dilutions were CFU-counted and added to a well plate that was placed into a plate reader to measure OD_600_ over time. From the growth curve data, the lag time can be extracted. It is critical to construct a new standard curve for each species under the exact conditions that will be used in the assay. This means preparing cells as they are prepared for the chemotaxis assay (see Material and Methods) and running the growth curve using the same settings and conditions that will be used when the assay is conducted.

**Fig 2:**
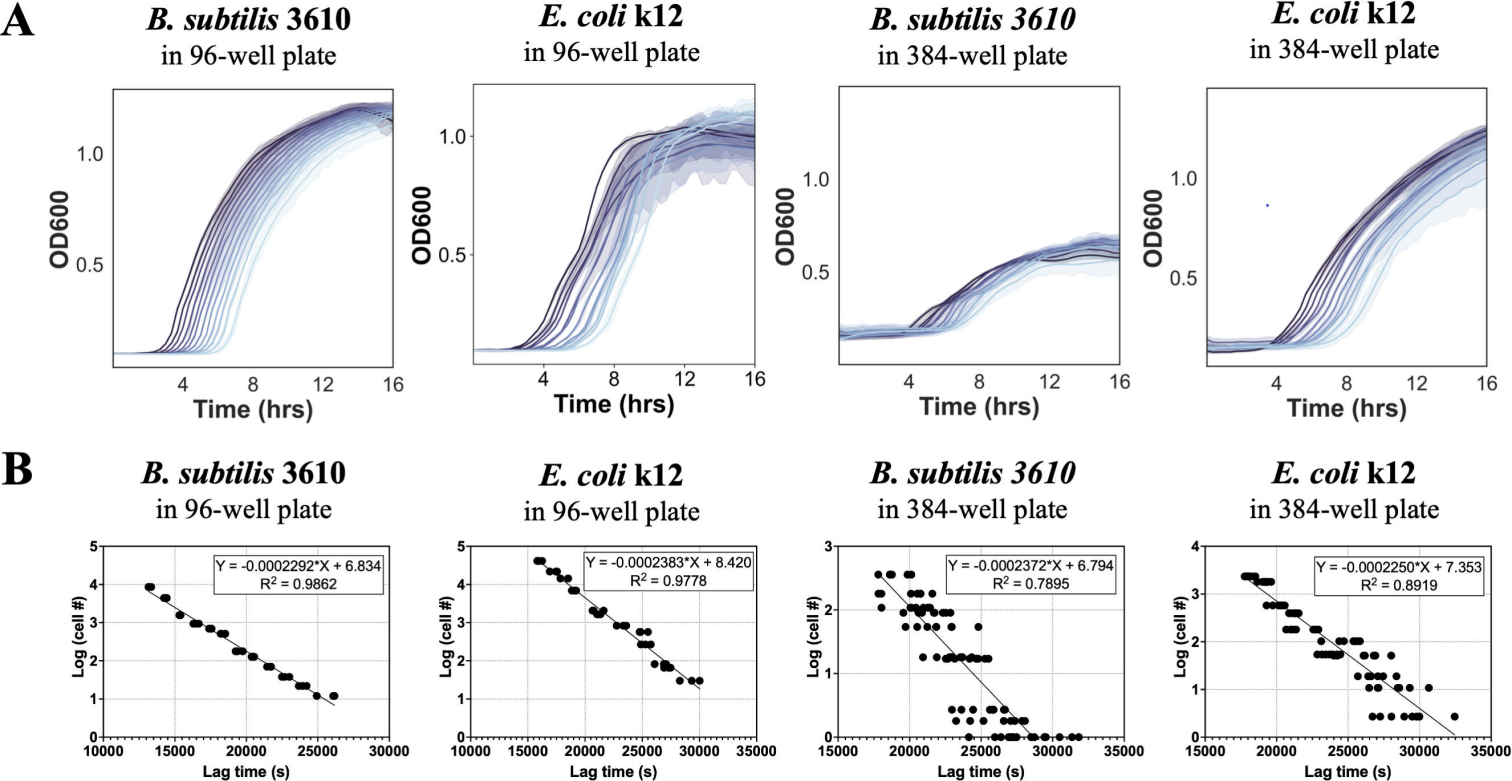
Constructing standard curves. Standard curves were constructed for each species in both 96- and 384-well plates by plotting the lag times of each well by the number of cells in each well. (**A**) Representative growth curves for the twofold serial dilutions of *Bacillus subtilis* 3610 and *Escherichia coli* K12 in 96- and 384-well plates. The solid lines indicate the mean of the technical replicates, while the shaded regions represent the 95% CI. Growth curves are colored by dilution with the wells with the highest concentration of bacterial cells shown in the darkest shade. For 96-well plates, three technical replicates were included for each condition. For 384-well plates, eight technical replicates were included for each condition. (**B**) Standard curves are calculated from growth curves by plotting the number of cells in each well against the lag time. Equations for the best fit line and *R*^2^ value are shown in the figure. Results in this figure are representative of two biological replicates, each containing three or eight technical replicates, for 96- or 384-well plates, respectively.

We constructed separate standard curves for *B. subtilis* and *E. coli* in both 96- and 384-well plates. For each standard curve, twofold serial dilutions were run in a plate reader; the resulting growth curves are shown in Fig. 2A, where each line represents a different dilution of cells. As the concentration of cells decreases, the lag time increases, resulting in a shifted growth curve. Growth dynamics of *B. subtilis* in the 384-well plate are noticeably different with a slower growth rate and lower maximum OD compared to the other graphs (Fig. 2A). We saw this consistently, highlighting the importance of constructing separate standard curves for each plate type. To calculate the standard curve, lag time is extracted from the growth curve data set and plotted against the number of cells in that well as determined by CFU counting (Fig. 2B). Using a simple linear regression, the slope of the standard line can be used in downstream experiments to estimate cell counts.

### 96-well capillary assay

The concentration of a chemoeffector at which no distinguishable difference from background can be measured is the threshold concentration. We were interested in how the sensitivity of our high-throughput method compares to the sensitivity of traditional quantitative capillary tube assays for *B. subtilis* and *E. coli*. For *B. subtilis*, the threshold concentrations for aspartate and serine were found to be 3 × 10^−5^ M and 6 × 10^−7^ M, respectively (10). The threshold concentration for *E. coli* toward aspartate and serine was identified to be 6 × 10^−8^ M and 3 × 10^−7^ M, respectively (9). To assess the sensitivity of our assay, we made 10-fold dilutions of serine and aspartate stock solutions and tested whether we could observe a difference between the number of cells in our control condition (0 M) compared to the different dilutions.

**Fig 3:**
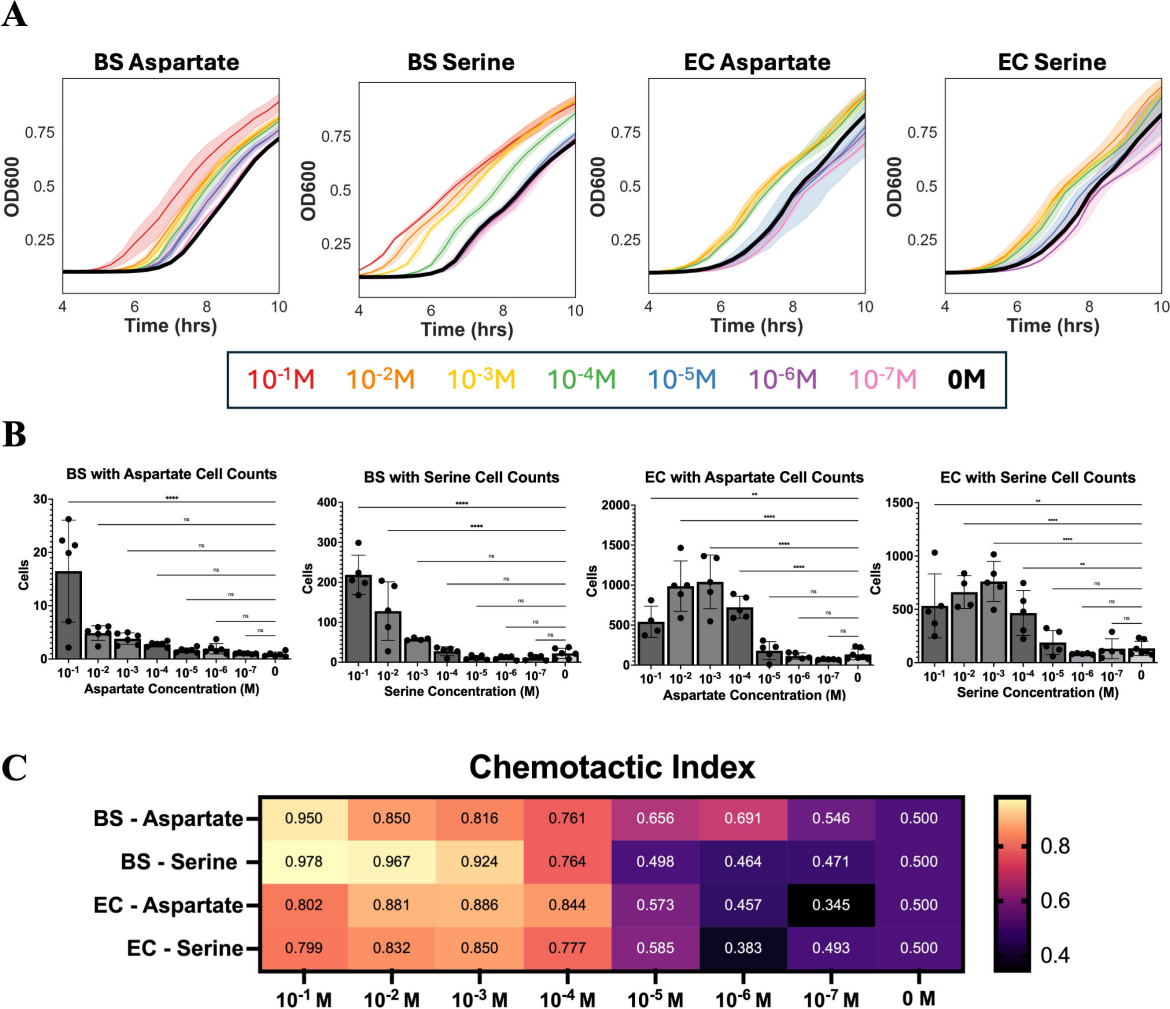
Ninety-six-well plate threshold concentrations. (**A**) Representative growth curve data from *B. subtilis* (BS) and *E. coli* (EC) chemotaxis experiments in 96-well plates. The solid lines indicate the mean of the six technical replicates, while the shaded regions represent the 95% CI. Growth curves are color coded by the concentration of the chemoeffector used in the sample. A legend for the color can be found below the graphs. (**B**) Calculated cell counts for *B. subtilis* and *E. coli* capillary tubes containing various concentrations of chemoeffectors. Cell counts are calculated from the growth curve data using the appropriate standard curve. Robust regression and outlier removal were used to remove outliers before plotting the data. Error bars denote a standard deviation of *n* = 6 technical replicates. One-way ANOVA with Dunnett’s test was used to calculate *P* values and compare each sample to the control. **, *P* < 0.01; ****, *P* < 0.0001; ns, *P* > 0.05. (**C**) Heat map showing the chemotactic index (CI) calculated for each condition. CI = *T* / (*T* + *C*), where *T* is the number of cells in the chemoeffector capillary, and *C* is the number of cells in the control capillary. CI > 0.6 is considered an attractant. Results in this figure are representative of two biological replicates, each containing six technical replicates.

All concentrations of aspartate tested for *B. subtilis* separated from the 0 M control growth curve (Fig. 3A). Cell counts remained low for *B. subtilis* toward aspartate. However, all dilutions of aspartate tested had average cell counts greater than the control data, with averages of 16.49, 4.88, 3.81, 2.74, 1.64, 1.92, 1.03, and 0.86 for aspartate concentrations of 10^−1^, 10^−2^, 10^−3^, 10^−4^, 10^−5^, 10^−6^, 10^−7^, and 0 M, respectively (Fig. 3B). The chemotactic index for the aspartate dilutions also highlights this decreasing trend, with 10^−1^ M aspartate having the highest CI value of 0.95 and 10^−7^ M having a CI value of 0.55, all other dilutions leading to CI values in between (Fig. 3C). Aspartate concentrations equal to or greater than 10^−6^ M have chemotactic indexes above 0.6, which indicates that they are attractants. Based on these data, we can conclude that the threshold concentration of aspartate chemoattraction in *B. subtilis* in our assay is 10^−6^ M.

In addition to aspartate, we also tested serine and found *B. subtilis* was highly responsive to concentrations of serine above 10^−4^ M. Concentrations below 10^−4^ M did not separate from the control growth curve (Fig. 3A) and had average cell counts equal to or slightly lower than the control condition (Fig. 3B). For the higher concentrations, cell counts were much higher than the cell counts for aspartate with averages of 119.02, 76.71, 32.12, 8.50, 2.60, 2.27, 2.34, 2.63 for serine concentrations of 10^−1^, 10^−2^, 10^−3^, 10^−4^, 10^−5^, 10^−6^, 10^−7^, and 0 M, respectively (Fig. 3B). From the chemotactic indexes shown in Fig. 3C, concentrations greater than or equal to 10^−4^ M serine can be considered attractant in our assay with CI values ranging from 0.764 to 0.978. Based on these data, we can conclude that the threshold concentration of serine chemoattraction in *B. subtilis* in our assay is 10^−4^ M.

For *E. coli*, concentrations of aspartate ranging from 10^−1^ to 10^−4^ M yielded similar results with overlapping curves (Fig. 3A). Unlike *B. subtilis*, for *E. coli*, the peak response was at 10^−3^ M rather than 10^−1^ M, with an average of 541.56, 989.44, 1,039.36, and 732.15 cells for aspartate concentrations of 10^−1^, 10^−2^, 10^−3^, and 10^−4^ M, respectively. After 10^−4^ M, there is a large decline in average cell counts with 179.42, 112.78, 70.62, and 133.85 cells in capillary tubes with aspartate concentrations of 10^−5^, 10^−6^, 10^−7^, and 0 M, respectively (Fig. 3B). Therefore, for these measurements, 10^−4^ M aspartate was found to be the threshold concentration for *E. coli*. This is also corroborated by the chemotactic index values in Fig. 3C, which fall between 0.802 and 0.886, indicating that these concentrations are effective chemoattractants.

Trends for *E. coli* chemotaxis toward serine are similar to the *E. coli* aspartate data. The threshold concentration of *E. coli* for chemotaxis toward serine is also 10^−4^ M, with concentrations below this resulting in overlapping growth curves with the motility media control (Fig. 3A). Like aspartate, responses to all concentrations of serine tested greater than 10^−4^ M were similar, with a peak response at 10^−3^ M (Fig. 3B). Concentrations above 10^−4^ M had high cell counts with averages of 531.90, 661.8, 761.06, and 465.84 for serine concentrations of 10^−1^, 10^−2^, 10^−3^, and 10^−4^ M, respectively (Fig. 3B). Concentrations of serine above 10^−3^ M had CI values ranging from 0.777 to 0.850, indicating attractants, while concentrations below this threshold had CI values ranging from 0.383 to 0.585, indicating little to no difference from the control.

### 384-well capillary assay

**Fig 4:**
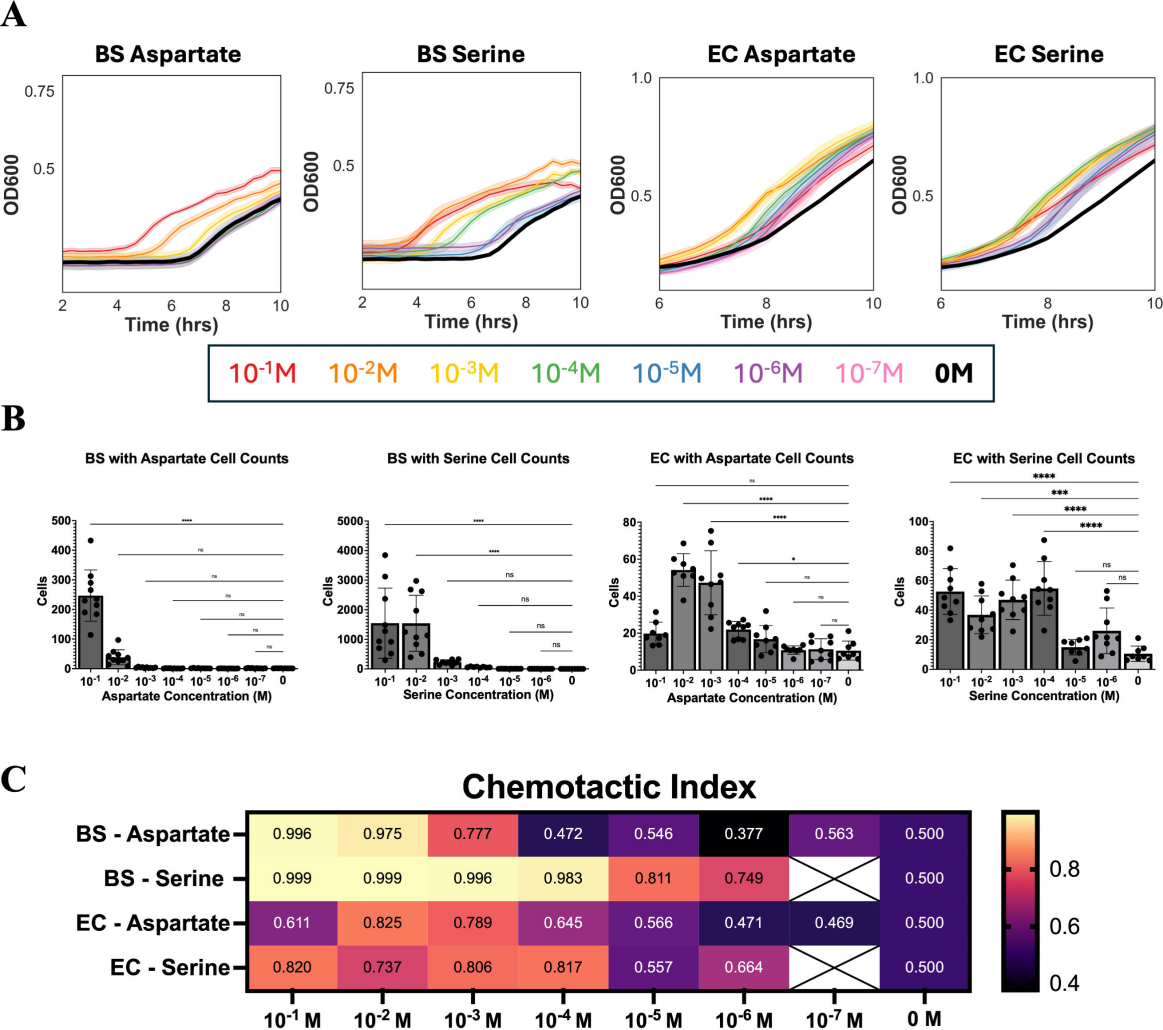
Three hundred eighty-four-well plate threshold concentrations. (**A**) Representative growth curve data from *B. subtilis* (BS) and *E. coli* (EC) chemotaxis experiments in 384-well plates. The solid lines indicate the mean of nine technical replicates, while the shaded regions represent the 95% CI. Growth curves are color coded by the concentration of the chemoeffector used in the sample. The legend for the color can be found below the graphs. (**B**) Cell counts are calculated from the growth curve data using the appropriate standard curve. Robust regression and outlier removal were used to remove outliers before plotting the data. Error bars denote a standard deviation of *n* = 9 technical replicates. One-way ANOVA with Dunnett’s test was used to calculate *P* values and compare each sample to the control. **, P < 0.01; ****, P < 0.0001; ns, *P* > 0.05. (**C**) Heat map showing the chemotactic index (CI) calculated for each condition. CI = *T* / (*T* + *C*), where *T* is the number of cells in the chemoeffector capillary, and *C* is the number of cells in the control capillary. CI > 0.6 is considered an attractant. Boxes with white X’s are samples for which data were not collected. Results in this figure are representative of two biological replicates, each containing nine technical replicates.

After optimizing this method using a 96-well plate format, we aimed to further increase the throughput by expanding to 384-well plates. To do this, we 3D printed a new capillary tube holder designed to accommodate the dimensions of Nunc 384-Well plates. We then validated the 384-well assay and investigated its sensitivity by repeating experiments done in the 96-well plates and comparing results. As seen in the 384-well standard curves, *B. subtilis* had stunted growth dynamics in the 384-well plate, with a slower growth rate and lower maximum OD when compared to *B. subtilis* grown in a 96-well plate (Fig. 4A). This was not the case for *E. coli*, which had a fast growth rate and maximum OD comparable to what is seen in growth in 96-well plates (Fig. 4A).

The 384-well plate successfully detected chemoattraction in all cases, where the concentration of the attractant was above 10^−3^ M. *B. subtilis* growth curves for different concentrations of aspartate above 10^−3^ M separated visibly on the growth curve, whereas for serine, all concentrations above 10^−6^ M separated visibly on the growth curve, suggesting a lower threshold concentration for serine compared to aspartate (Fig. 4A). In general, serine elicited a stronger response from *B. subtilis* than aspartate with the highest cell counts for serine capillaries in the 10^−1^ M dilution with 1,545.32 cells, while the highest response for aspartate was in the 10^−1^ M dilution with 246.83 cells (Fig. 4B). The stronger response of *B. subtilis* toward serine was also reflected in the CI values for the lower concentrations, which were all greater than 0.7, indicating a chemoattraction (Fig. 4C). For aspartate, the threshold was higher at 10^−3^ M with a CI value of 0.77 (Fig. 4C).

*E. coli* data collected using the 384-well plate closely resembled the data collected using our 96-well plate with threshold concentrations for both serine and aspartate at 10^−4^ M. For aspartate, *E. coli* peak response was found to be 10^−2^ M, while for serine, it was 10^−1^ M. *E. coli* growth curves for the dilutions of aspartate and serine separated from the control but were more overlapping than the *B. subtilis* curves. This may indicate a non-linear response of *E. coli* to the different concentrations of chemoeffectors (Fig. 4A). *E. coli* showed similar magnitudes of response to both aspartate and serine, with peak cell counts at 54.6 and 52.9 for aspartate and serine, respectively (Fig. 4B). CI values for *E. coli* indicate that all concentrations greater than or equal to 10^−4^ M for both chemoeffectors are chemoattractants (Fig. 4C).

## DISCUSSION

In this study, we optimized a high-throughput capillary tube-based chemotaxis assay to allow for rapid screening of chemoattractants. We demonstrated that the system is effective for studying both gram-negative and gram-positive organisms and that the assay is functional in both 96- and 384-well formats. This greatly expands on the current capabilities of the field, describing for the first time a method to simultaneously run up to 384 capillary tube assays, enabling researchers to expand the libraries being tested when describing chemotactic profiles of bacteria of interest.

The throughput of the assay relies upon the relationship between lag time and starting cell concentration. This allows for the use of growth curves in plate readers for data collection and dramatically reduces the workload and user error associated with traditional quantification strategies such as CFU counting. The assay can be adapted to work with any plate layout that can be read using a plate reader. We believe that the throughput could be further expanded to include 1,536-well plates. The reliance on plate readers for quantification, however, does introduce some limitations. It requires that the bacteria being studied is compatible with plate-based growth in a manner in which the linear relationship between lag time and OD is maintained. Additionally, sources of error inherent to the plate reader become relevant considerations here. This includes the influence of evaporation, which may cause edge effects that may become more prominent when using smaller well volumes or running growth curves for prolonged periods of time. This may be avoided by filling edge wells with water rather than samples.

The sensitivity of our assay was investigated for both the 96- and 384-well layouts. We found that for the 96-well plate, threshold concentrations for *B. subtilis* toward aspartate and serine were 10^−6^ and 10^−4^ M, respectively. This is aligned with previously reported threshold concentrations (10). For the 384-well plates, the threshold concentrations were 10^−3^ and 10^−7^ M for aspartate and serine, respectively. Discrepancies in these values may be due to the physical differences in the well plates, as *B. subtilis* exhibited altered growth behavior in the 384-well plate, likely due to reduced oxygen diffusion in the smaller wells of the 384-well plate. This reduced the fit of our standard curve, resulting in a lower *R*^2^ value compared to the 96-well plate. This likely impacted the accuracy of our cell count calculations for the 384-well plate, suggesting that in some cases, there may be a trade-off between throughput and sensitivity if lower chemoattractant concentrations are being studied. This is a limitation of the assay, highlighting the dependence of this technique on a linear relationship between cell count and lag time, which may be dependent on the species and the specific conditions of the assay.

For *E. coli*, the threshold concentrations for aspartate and serine were consistently at 10^−4^ M, irrespective of whether the 96- or 384-well plate was used. These threshold values for *E. coli* are higher than what was previously reported for manual capillary tube assays (9). This discrepancy may be because the referenced paper performed the assay at 30°C, which has been shown to increase *E. coli* cell motility (7), while the incubation part of our assay was conducted at room temperature. However, we show that the assay can detect chemoattraction toward concentrations that span many magnitudes of order, which should be sufficient for most high-throughput screening purposes. Additional limitations to this assay exist that are inherent to all capillary tube assays. This includes the requirement of swimming motility and the inability to draw conclusions about chemorepulsion. Additional work is needed to develop a high-throughput assay to screen bacterial chemorepellents and bacteria that use twitching- or swarming-based motility.

In summary, here, we have described a novel 3D-printed capillary tube holder that can be used to conduct up to 384 individual capillary tube assays simultaneously. We show that our system is effective in both the 96- and 384-well formats and that it is sufficiently sensitive to detect changes in chemotactic behavior against a gradient of chemoeffector concentrations in both gram-negative and gram-positive bacteria. This method greatly reduces the time required to screen large libraries of chemoeffectors and will enable the generation of larger data sets characterizing bacterial chemotactic behavior.

## MATERIALS AND METHODS

### Materials

Fisherbrand 96-Well plates (Cat. No. FB012931) and Nunc 384-Well plates (Cat. No. 242757) were used for our assays along with 1 µL capillary tubes from Drummond (Cat. No. 1-000-0010) and TempPlate XP sealing film (USA Scientific #2972-2100).

### Designing and printing the 3D capillary tube holder

Designs were created using OnShape and printed at the Caltech TechHub on a Bambu X1 Carbon printer using TPU filament (ZIRO TPU Filament 1.75 mm, Shore 95A). The .stl file was sliced for printing using the Orca slicer software; 0.20 mm standard settings were used with slight modifications: bed type = “smooth high temp plate” and filament type = “generic TPU.” For new prints, we recommend going through each hole with a 20-gage needle (or needle with an outer diameter closest to the desired hole diameter) before trying to insert the capillaries. The capillaries should fit tightly but should be inserted and removed without breaking. For optimization, a calibration print is included in File S1, which can be used to determine the optimal capillary hole diameter based on the print conditions. Print files for the calibration piece and 96-well capillary holder can be found in File S1 and S2; see File S3 at https://doi.org/10.5061/dryad.2jm63xt2v.

### Testing capillary expulsion speed

Capillary tube holders were filled with empty capillary tubes and placed atop a well plate containing food coloring. The capillaries were allowed to fill with food coloring before being moved to an empty plate. The capillary tube holder on the empty plate was placed in an Eppendorf Centrifuge 5910 Ri with a second 3D-printed capillary tube holder acting as a balance. Various spin settings were tested for complete expulsion, which was confirmed by observing the remaining food coloring in the capillary tubes after spinning.

### Preparing bacterial cells for experiments

*E. coli* K12 (ATCC 25404) and *B. subtilis* 3610 (ATCC 6051) were inoculated into LB media and incubated at 37°C with shaking at 250 rpm for 16–18 hours. Overnight cultures were diluted approximately 100-fold in fresh LB media and grown at 37°C with shaking at 250 rpm until the mid-exponential phase (OD_600_ of 0.35–0.6) to ensure high motility and cell viability before assay preparation. The volume of each culture needed to obtain 1.5 mL of cells at an OD_600_ = 1.0 was then calculated from the measured OD_600_. The corresponding volume of overnight culture was centrifuged for 5 min at 1,700 × *g*, and the supernatant was removed. The pellets were washed with motility media (10 mM potassium phosphate buffer, pH 7; 0.1 mM EDTA; and 0.05% glycerol) and resuspended by gentle inversion to avoid disrupting motility machinery. This wash step was repeated again under the same conditions. Following the second wash, the cultures were centrifuged with the same parameters and resuspended in 1 mL of motility media. OD_600_ was checked following washing, and the cell suspensions were diluted to the desired OD_600_.

### Constructing standard curves

*E. coli* and *B. subtilis* cultures were prepared and washed as described in Preparing Bacterial Cells for Experiments. After the final resuspension, the cultures were diluted with LB to an OD_600_ of 10⁻⁴ from which 11 twofold serial dilutions in LB were made. From the serially diluted cultures, 180 µL was added to each of the designated wells of a 96- or 384-well plate, with at least three replicates for each concentration of cells. The plate was placed in a Tecan Infinite M Nano plate reader for 24 hours at 37°C and with continuous orbital shaking at ~200 rpm, recording OD_600_ at 20-min intervals. CFU counting of each serially diluted culture was used to determine the number of cells in each well. The standard curves were calculated from the resulting growth curves by plotting the number of cells in each well against the lag time (time required for each well to reach an OD of 0.3).

### 96-well plate chemotaxis assay

*E. coli* and *B. subtilis* cultures were prepared and washed as explained in Preparing Bacterial Cells for Experiments. The cell suspensions were diluted with motility media to a final OD_600_ of 0.01. A total of 180 μL of each diluted culture was then added to the designated wells of a 96-well plate. The plate was then allowed to incubate statically at 37°C for 30 min to allow cells to recover after washing.

To prepare the chemoeffector plate, 180 μL of seven 10-fold serially diluted concentrations (10⁻¹, 10⁻², 10⁻³, 10⁻⁴, 10⁻⁵, 10⁻⁶, and 10⁻⁷ M) of serine and aspartate was added to their designated wells, with motility media in control wells. A wash plate was prepared by adding 200 μL of sterile ddH_2_O water to each well of a third 96-well plate. After the bacterial plate had finished recovering, the capillary holder containing 1 µL capillaries in each opening was placed on top of the chemoeffector plate. The capillary holder was left on for at least 2 min to ensure each of the capillaries filled. The top of the capillary tube holder was sealed tightly with plate film, transferred to the wash plate, and dipped up and down three times to remove excess chemoeffector solution on the outside of the capillaries. The holder was then transferred to the bacterial plate and incubated at room temperature for 45 min. Following incubation, the capillary tube holder containing the capillaries was placed on a blank plate and centrifuged for 2 min in an Eppendorf Centrifuge 5910 Ri at 1,000×*g* to expel their contents. After centrifugation, the capillary tube holder was removed, and a total of 180 μL of LB media was added to each well. The plate was then placed in a Tecan Infinite M Nano plate reader for 24 hours at 37°C and with continuous orbital shaking at ~200 rpm, recording OD_600_ at 20-min intervals.

### 384-well plate chemotaxis assay

*E. coli* and *B. subtilis* cultures were prepared and washed as explained in Preparing Bacterial Cells for Experiments. The cell suspensions were diluted with motility media to a final OD_600_ of 0.01. With each of the diluted cultures, 60 µL was added to the designated wells of a 384-well plate. The plate was then allowed to incubate statically at 37°C for 30 min to allow cells to recover after washing.

The chemoeffector plate was then prepared by adding 60 μL of seven 10-fold serially diluted concentrations (10⁻¹, 10⁻², 10⁻³, 10⁻⁴, 10⁻⁵, 10⁻⁶, and 10⁻⁷ M) of serine and aspartate to their designated wells, with motility media in control wells. A wash plate was prepared by adding 80 μL of sterile ddH_2_O water to each well of a third 384-well plate. The steps for filling, washing, incubating, and expelling the capillaries were performed as described in 96-Well Plate Chemotaxis Assay, except that a capillary holder with 384 openings was used in place of the 96-well holder. After the capillary contents were expelled, a total of 90 μL of LB medium was added to each well, and the plate was placed in a Tecan Infinite M Nano plate reader for 24 hours at 37°C and with continuous orbital shaking at ~200 rpm, recording OD_600_ at 20-min intervals.

## Data Availability

Supplemental File S3 can be accessed at https://doi.org/10.5061/dryad.2jm63xt2v.

## SUPPLEMENTAL MATERIAL

File S1 jb.00384-25-s0001.stl Calibration print file that can be printed to determine the optimal diameter for the holes in the capillary tube holder.

File S2 jb.00384-25-s0002.stl Design file to print the 96 well capillary tube holders.

ASM does not own the copyrights to Supplemental Material that may be linked to, or accessed through, an article. The authors have granted ASM a non-exclusive, world-wide license to publish the Supplemental Material files. Please contact the corresponding author directly for reuse.
